# A High-Sensitivity Resonant Magnetic Sensor Based on Graphene Nanomechanical Resonator

**DOI:** 10.3390/mi13040628

**Published:** 2022-04-16

**Authors:** Wenyao Liu, Wei Li, Chenxi Liu, Enbo Xing, Yanru Zhou, Lai Liu, Jun Tang

**Affiliations:** 1 Shanxi Province Key Laboratory of Quantum Sensing and Precision Measurement, North University of China, Taiyuan 030051, China; liuwenyao@nuc.edu.cn (W.L.); b200609@st.nuc.edu.cn (W.L.); s202106138@st.nuc.edu.cn (C.L.); xiaoxing@126.com (E.X.); zhouyanru@nuc.edu.cn (Y.Z.); liulaiopto@163.com (L.L.); 2 State Key Laboratory of Dynamic Measurement Technology, North University of China, Taiyuan 030051, China

**Keywords:** nanomechanical resonator, graphene, magnetic sensor

## Abstract

This paper presents a novel resonant magnetic sensor consisting of a graphene nanomechanical oscillator and magnetostrictive stress coupling structure, using Si/SiO_2_ substrate and Fe–Ga alloy, respectively. In this device, the deformation of the Fe–Ga alloy resulting from the external magnetic field changed the surface tension of the graphene, resulting in a significant change in the resonance frequency of graphene. Using the finite element analysis, it could be found that the response of the resonance frequency revealed a good linear relationship with the external magnetic field (along the *x*-axis) in the range of the 1 to 1.6 mT. By optimizing the sizes of each component of the magnetic sensor, such as the thickness of the Si/SiO_2_ substrate and the Fe–Ga alloy, and the length of the graphene, the sensitivity could even reach 834 kHz/mT, which is three orders of magnitude higher than conventional resonant magnetic devices. This provides a new method for highly sensitive and miniaturized magnetic sensors.

## 1. Introduction

Magnetic sensors have a wide range of applications in different areas such as navigation, detection, and medicine [1,2,3]. Resonant magnetic sensors based on microelectromechanical systems (MEMS) technology have attracted the extensive attention of scholars due to their advantages of small size, light weight, and low cost [4]. At present, the mainstream solution for resonant magnetic sensors is to process the resonant structure on a silicon wafer, and then use the Lorentz force generated by the interaction between the magnetic field and the current-carrying structure to drive the structure to deflect. Generally, the deflection is detected by optical [5] or electrical [6] means. Due to the high applied current required to drive the resonant structure, it has disadvantage of high power consumption.

In recent years, magnetostrictive materials have attracted increasing attention as a class of alloy materials whose dimensions can be changed in response to external magnetic fields. Bian et al. [7] designed a resonant magnetic sensor based on a piezoelectric quartz crystal double-ended tuning fork (DETF). The magnetostrictive force was converted into longitudinal force to change the resonant frequency of DETF. The measured sensitivity in the linear region was 35 Hz/mT. In addition, this group designed a resonant magnetic sensor with a bias magnetic circuit by combining a magnetostrictive material with a quartz tuning fork, achieving a sensitivity of 26 Hz/mT in the range of 5 mT [8]. These systems are simple in structure and easy to drive. However, the resonant frequency of the resonant structure needs to be further improved, which is a key factor affecting the sensitivity of the magnetic sensor. 

As a new two-dimensional ultrathin nanomaterial, graphene has rapidly attracted the attention of experts and scholars in the sensor field due to its outstanding mechanical and electrical properties. Subsequently, a series of studies have been carried out on the resonant effect of graphene and related devices. At present, scholars at home and abroad have developed resonant nanoelectromechanical systems (NEMS) based on single-layer and multilayer graphene, and they have explored the resonance characteristics of graphene from the perspectives of experiments and simulations, confirming its feasibility as a resonator [9,10,11,12,13]. Subsequently, graphene-based resonant pressure sensors [14,15,16], resonant mass sensors [12,17,18,19,20], and resonant acceleration sensors [21,22,23] have been extensively investigated by scholars through elastic mechanics theory and molecular dynamics simulations. However, graphene-based resonant magnetic sensors are still rare. 

In this work, a NEMS resonant magnetic sensor of graphene based on the magnetostrictive effect was designed. The displacement distribution, as well as the maximum stress and strain components of Fe–Ga alloy, as a function of the magnetic field under magnetic field modeling was analyzed. Then, the sizes of each component were optimized using finite element simulation with magnetostrictive theory. In addition, this device is compatible with semiconductor technology, showing good application potential.

## 2. Modeling Analysis of Magnetic Sensor

**Figure 1:**
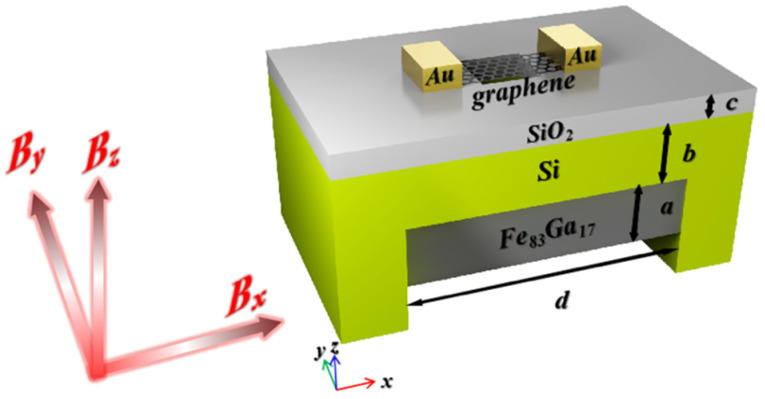
Schematic diagram of a graphene magnetic sensor chip.

The schematic diagram of the graphene magnetic sensor chip including a graphene resonator, a Si/SiO_2_ substrate, and an iron–gallium alloy is shown in Figure 1. In our model, the magnetostrictive material under the groove was the sensitive element of the resonator, and the most widely used Fe–Ga alloy (Fe_83_Ga_17_) was chosen in this model as the material with the characteristics of a large magnetostriction coefficient, high strength, excellent processing performance, and high magnetic permeability under a low-saturation magnetic field [24,25]. The parameters of the Fe–Ga alloy and graphene are shown in Table 1.

Compared to rare-earth magnetostrictive materials (Terfenol-D) and piezoelectric materials, Fe–Ga alloys have good mechanical properties such as low brittleness and high tensile strength, which can be produced by magnetron sputtering [28,29]. They have good compatibility with semiconductor technology. In order to increase the adhesion between graphene and the substrate, a 2 μm thick SiO_2_ layer was evaporated between them as an insulating layer, and a square cavity with a side length of 2~10 μm was etched in the center of the SiO_2_ insulating layer. A doubly clamped graphene beam was suspended on the surface of the cavity. Gold electrodes were deposited on both sides of graphene and bonded with peripheral circuits to drive the resonator through electrical modulation. The readout of the graphene resonant NEMS can be detected using optical interference [30], all-electric mixing frequency modulation [31,32,33], or atomic force microscopy [34]. The fundamental resonance frequency for a doubly clamped beam is given by:

(1) $$f(0)=A\sqrt{\frac{E}{\rho }}\frac{t}{L^{2}}$$

where, *A* is clamping coefficient, *E* is the Young’s modulus of the resonator, *ρ* is the density of the resonator, *t* is the thickness of the resonator, *L* is the length of the resonator suspended on the cavity.

The Fe–Ga alloy produces a positive stretching along the direction of the magnetic field under an external magnetic field. The deformation of the substrate causes the two constrained edges of the graphene to generate tensile stresses in opposite directions, changing the resonance frequency of the graphene. Then, the intensity of the magnetic field can be obtained by measuring the change in resonance frequency. 

Generally, when the variation range of magnetic field intensity is small, the relationship between magnetic stress and magnetic field can be simply expressed as *α_B_B*, where *α_B_* and *B* are the magnetostrictive coefficient and magnetic field intensity of the Fe–Ga alloys, respectively. The thickness of SiO_2_ is less than that of Si; thus, the stress conducting layer is approximately considered as a Si layer. The stress acting on the resonator is

(2) $$\sigma_{x}=\gamma \sigma_{B}=\frac{6(1+\upsilon_{\mathrm{Si}})}{\pi^{2}}{\left(\frac{d}{2b+2c}\right)}^{2}\gamma \alpha_{B}B,$$

where *γ* is the transfer coefficient of the stress on the substrate to the constrained edge of the resonator, *ν*_Si_ = 0.22 is the Poisson’s ratio of Si, *a* = 5 µm is the thickness of the Fe–Ga alloy, *b* = 4 µm is the thickness of Si, *c* = 2 µm is the thickness of SiO_2_, and *d* = 60 µm is the axial length of Si cavity. The fundamental resonance frequency for a resonator under axial stress $\sigma_{x}$ is expressed as follows [14]:

(3) $$f(\sigma_{x})=f(0)\sqrt{1+\frac{\sigma_{x}}{\sigma_{c}}},$$

(4) $$\sigma_{c}=\frac{\pi^{2}Et^{2}}{3L^{2}}.$$

The frequency caused by stress can be expressed as

(5) $$f(\sigma_{x})=A\frac{t}{L^{2}}\sqrt{\left(\frac{E}{\rho }\right)\cdot \left(1+\frac{3(1+\upsilon_{\mathrm{Si}})d^{2}}{2\pi^{2}{\left(b+c\right)}^{2}}\cdot \frac{3\gamma \alpha_{B}BL^{2}}{\pi^{2}Et^{2}}\right)},$$

where $\sigma_{c}$ is Euler’s critical buckling load, and *f*($\sigma_{x}$) is the frequency after magnetic field loading. The material parameters are as follows: *t* is the thickness of graphene, *L* = 10 µm is the length of graphene suspended on the cavity, and *W* = 2 µm is the width of graphene, which is close to the previous parameters [30]. *E* is the Young’s modulus of graphene, and *ρ* is the density of graphene. 

As can be seen from Equation (5), when the main structure is determined, the resonance frequency of graphene is related to the length, thickness, magnetic field intensity, angle, etc. Therefore, in order to investigate the frequency scaling of graphene in this structure, we used FEM (finite element method) to compute the resonators. Then, we optimized the key parameters affecting the sensing performance to further improve the directivity and sensitivity of the sensor.

## 3. Results and Discussion

In order to explore the performance of the magnetic sensor, graphene and substrate were regarded as a shell structure with conventional solid mechanics in COMSOL Multiphysics. We used the well-known value of 0.335 nm for the thickness of the single-layer graphene sheets and a Poisson’s ratio of 0.16. This thickness provided a Young’s modulus on the order of 1 TPa. Shell–solid coupling was used to connect the shell and the solid. The lower surface of the substrate was fixed, and *B* indicates the external magnetic field along the *x*-axis, *y*-axis, and *z*-axis.

**Figure 2:**
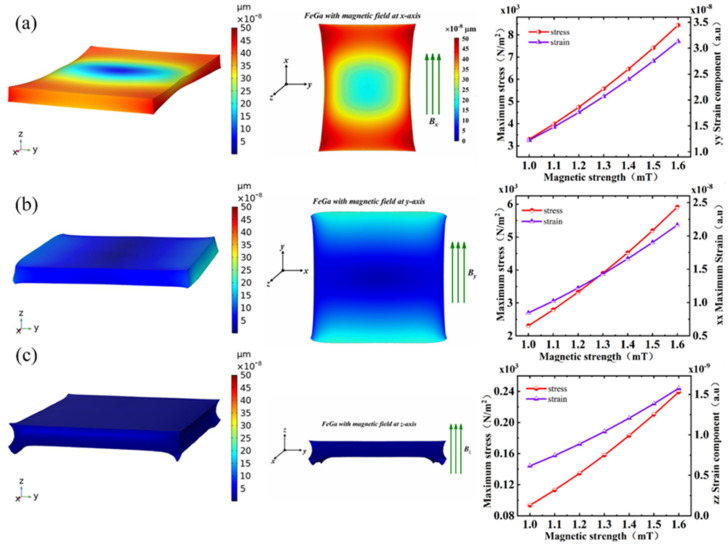
The 3D displacement distribution of Fe–Ga alloy under 1 mT magnetic field (**left**), 2D displacement distribution of Fe–Ga alloy under 1 mT magnetic field (**middle**), and its maximum stress and strain components as a function of the magnetic field (**right**). The magnetic field is shown along the (**a**) *x*-axis, (**b**) *y*-axis, and (**c**) *z*-axis.

**Figure 3:**
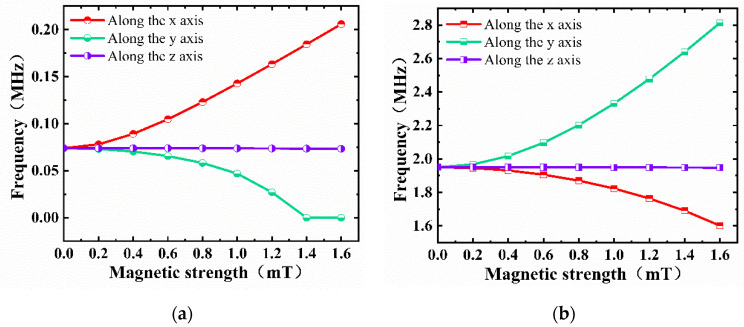
Resonance frequency versus magnetic field in different directions when (**a**) beam is clamped on two edges, and (**b**) beam is clamped on four edges.

Figure 2 shows the characteristic curves of stress and strain as a function of the magnetic field in Fe–Ga alloy. It can be seen that the deformation in all directions was different due to the different spatial orientation of alloy magnetic domains. When the direction of the magnetic field was along the *x*-axis, the maximum stress and maximum strain components of the Fe–Ga alloy were largest, compared with the other two directions. On the contrary, they were not obvious along the *z*-axis. When the magnetic field was along the *x*-axis, maximum strain components could reach 1.3–3.5 × 10^−8^, and the corresponding stress reached 3–9 × 10^3^ N/m^2^. Furthermore, when the magnetic field increased from 1 mT to 1.6 mT, the maximum stress and strain components showed a quasi-linear increasing trend. This is also critical to the linearity of the sensor. The resonance frequency versus magnetic field in different directions is also shown in Figure 3.

When the two edges were clamped, the resonance frequency as a function of magnetic field was as shown in Figure 3a. The resonance frequency increased with the increase in magnetic field when the direction of the magnetic field was along the *x*-axis, whereas the resonance frequency decreased with the increase in the magnetic field and eventually approached 0 when the direction of the magnetic field was along the *y*-axis. It can be seen that the resonance frequency hardly changed when the direction of the magnetic field was along the *z*-axis. When the four edges were clamped, as shown in Figure 3b, the trends were generally opposite to those seen in Figure 3a.

We can find that the resonance frequency increased with the increase in magnetic tension, whether it was clamped on two sides or four sides. Moreover, when the four sides were clamped, the frequency was about 25 times higher than that when two sides were clamped [10]. When two sides of graphene were clamped along the *y*-axis, magnetic stress was applied in the *x*-axis direction, which led to an increase in the tension of graphene and, thus, a gradual increase in its resonant frequency. When the magnetic field was applied in the *y*-axis direction, graphene was not fixed in this direction, which made it difficult to influence the resonance frequency in theory. However, with the increase in stress in the *y*-axis direction, the resonance of the film in the *x*-axis direction could be suppressed, resulting in a decrease in frequency. When all four sides were clamped, the resonance mode caused by the magnetic field was different from that when two sides were clamped, and the deformation caused by stress affected the resonance frequency. Especially when the aspect ratio of graphene was large, the long side was more likely to cause deformation than the short side, thus presenting a different phenomenon compared to when two sides were clamped.

Although the structure clamped on four edges showed a higher frequency, the linearity with the magnetic field was relatively poor. Thus, we mainly discuss when the beam was clamped on two edges below. Accordingly, the magnetic field along the *x*-axis was taken as an example to study in detail.

**Figure 4:**
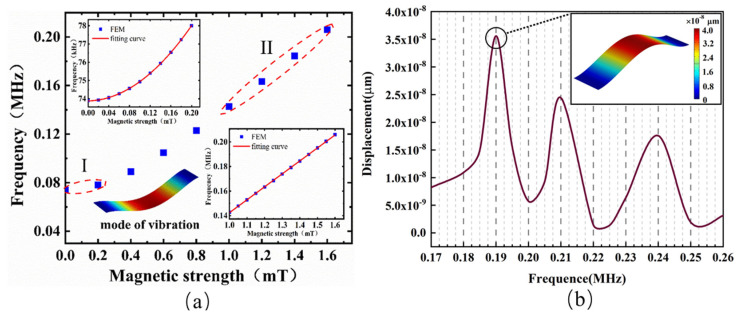
(**a**) Resonance frequency versus external magnetic field in the range of 1–1.6 mT. The inset shows the nonlinear change at 0–0.2 mT (I) and the linear change at 1–1.6 mT (II); (**b**) amplitude versus frequency for graphene resonator when the magnetic field was 1.4 mT. (Inset) Free vibration mode shapes of graphene sheets.

Figure 4a shows the influence of changes in the external magnetic field (0–1.6 mT) on the first-order fundamental resonance frequency of the mechanical oscillator. As the magnetic field increased, the resonance frequency also increased. By fitting the characteristic points in the range of 0 to 0.2 mT and 1 to 1.6 mT, it was found that the fitting curve followed a nonlinear change in area I and a linear change in area II. This may have been due to the nonlinear magnetostrictive effect of the Fe–Ga alloy under a weak magnetic field in the range of 0–0.2 mT, which caused the tensile stress on the constrained edges of the graphene to change nonlinearly, resulting in a nonlinear change in the resonance frequency with the magnetic field. When the magnetic field *B*_0_ > 1 mT, the Fe–Ga alloy had an approximately linear magnetostrictive effect, which ultimately caused the resonance frequency to change linearly with the magnetic field. The sensitivity of the magnetic sensor was about 105 kHz/mT according to an estimation of the slope of the fitting curve of the area II, which is at least three orders of magnitude higher than previous resonant magnetic sensors based on the magnetostrictive effect [7,8].

Then, the frequency characteristics were also investigated, using the frequency-domain function of COMSOL. The sweep frequency range was 0.15 to 0.30 MHz, and the step size was 0.005 MHz The analysis point here is located in the center of graphene sheets. As shown in Figure 4b, the first resonance peak was found at 0.19 MHz, which is basically consistent with the steady-state vibration frequency calculation. The full width at half maximum (FWHM) of the fundamental resonance was 6.7 kHz, and the calculated Q factor was about 28. Bunch et al. reported single-layer graphene clamped on two sides with a size of about 5 μm × 2 μm and a fundamental mode Q value of 78 [30]. By comparison, we found that the calculated Q factor and the resonance frequency were lower than the experimental value. This may have been related to the increase in tension in the production of single-layer graphene. In addition, the Q factor was not only related to the size of graphene, but also related to the excitation mode, resonance frequency, testing environment, and adsorption with the substrate.

**Figure 5:**
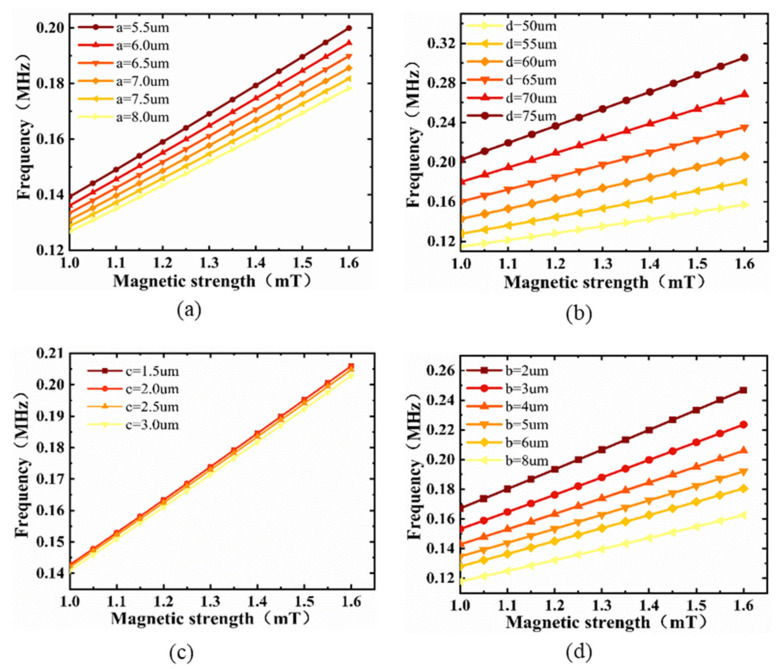
Resonance frequency versus external magnetic field in the range of 1–1.6 mT for various sizes of (**a**) Fe–Ga alloy, (**b**) groove, (**c**) SiO_2_, and (**d**) Si.

The Si/SiO_2_ substrate is a key component for conducting stress, and it is crucial to study the effect of its size on the resonance frequency of the magnetic sensor. Figure 5a,b show the resonance frequency as a function of the thickness of the Fe–Ga alloy and the width of the groove, respectively. The sensitivity increased slightly with the increase in thickness and width, while the resonance frequency also increased, and the six curves of the two figures showed a good linear relationship. Figure 5c shows the resonance frequency as a function of the thickness of SiO_2_. In the actual manufacturing process, since the thickness of SiO_2_ is generally a few microns or even nanometers, the difference in the thickness of the SiO_2_ was set to 0.5 µm in the finite element simulation. It was found that, with the increase in thickness, the increase in resonance frequency and sensitivity was almost negligible, and the six curves almost overlapped, also owing to the small difference in thickness of SiO_2_. In order to study the influence of the thickness of the substrate on the resonance frequency further, we simulated the curve of the resonance frequency by changing the thickness of Si, as shown in Figure 5d. The resonance frequency decreased with the increase in thickness of Si, and the sensitivity also slightly decreased. Combined with the above analysis, selecting the substrate size as a = 5.5 µm, b = 2 µm, c = 1.5 µm, d = 75 µm could obtain the highest resonance frequency.

**Figure 6:**
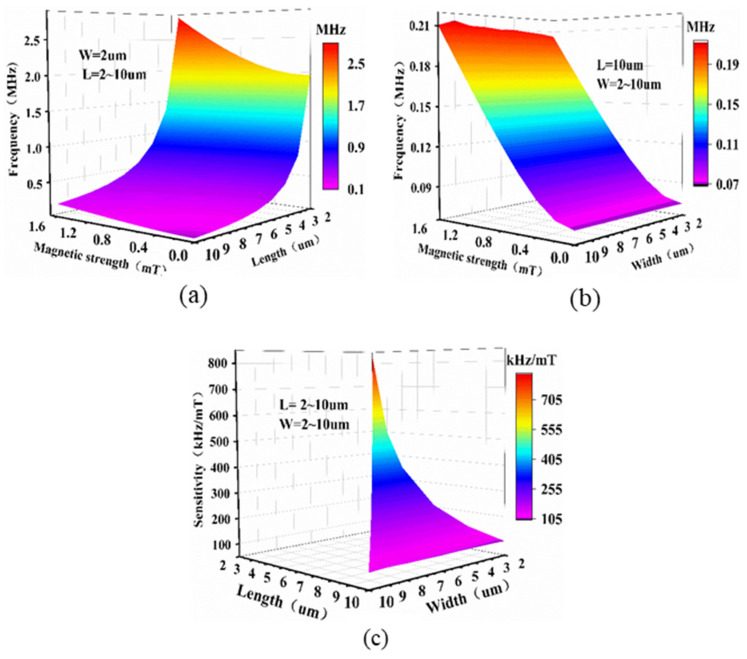
Resonance frequency versus magnetic field as a function of (**a**) length, and (**b**) width. (**c**) Sensitivity of beam as a function of length and width.

In order to study the influence of the size of the graphene on the magnetic sensor, the influence of the length, width, and thickness of the membrane on its resonance frequency were simulated, as shown in Figure 6a,b. When *W* = 2 µm, the resonance frequency increased gradually with decreasing length, and the rate of increase gradually became larger. This is consistent with the description of Equation (5). When *L* = 2 µm, *W* = 2 µm, the resonance frequency was greater than 2.5 MHz under an external magnetic field of 1.6 mT. When *L* = 2 µm, the resonance frequency hardly changed with the increase in width. It can be seen that the *L* of the membrane had a greater impact on its resonance frequency, and the changes in *W* hardly affected the resonance frequency. In order to obtain sensitivity as a function of length and width, a series of points with *L* and *W* (*L ≥ W*) between 2 µm and 10 µm were simulated. Figure 6c shows its three-dimensional mapping surface. Larger membranes exhibited smaller sensitivity. When the size of the membrane was 2 µm × 2 µm, the sensitivity was about 834 kHz/mT.

**Figure 7:**
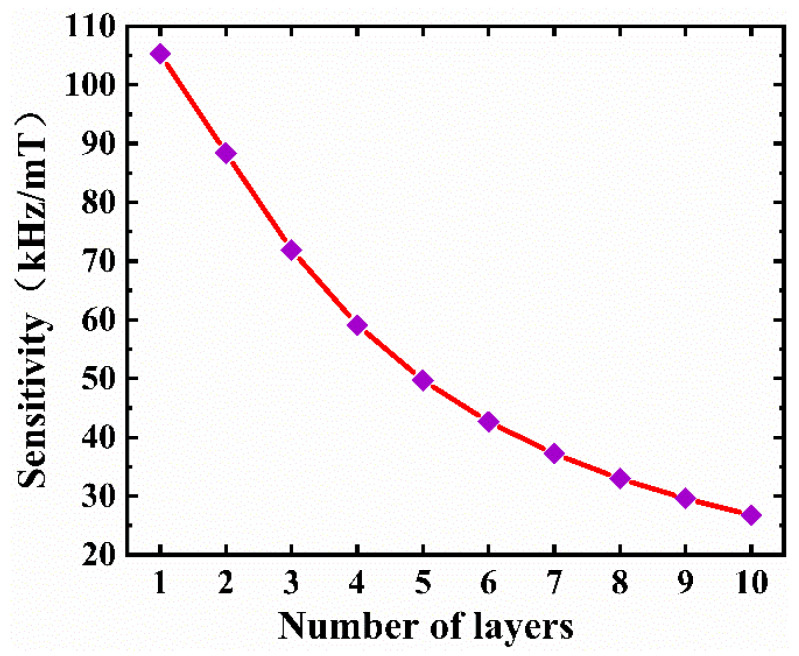
Variation of the sensitivity of the magnetic sensor with the number of graphene layers.

The effect of thickness on sensitivity is plotted in Figure 7. The sensitivity dramatically decreased with increasing number of layers and achieved its maximum for the single-layer graphene. In the actual manufacturing process, selecting graphene with a small size and thickness can obviously maximize the sensitivity of the magnetic sensor.

**Figure 8:**
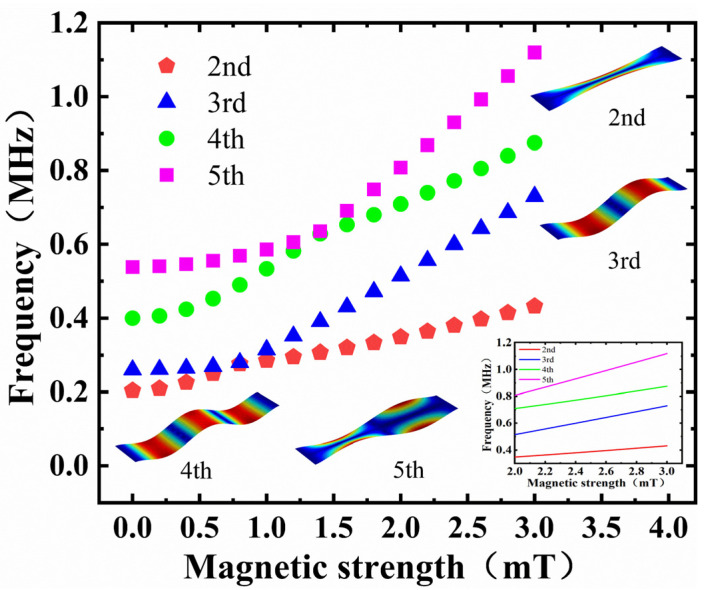
The second to fifth natural frequencies of the magnetic sensor changes versus the magnetic field. The inset shows the fitted curve of 2–3 mT.

In order to obtain the relationship between the natural frequencies of different orders and the external magnetic field (0–3 mT), the second to fifth natural frequencies of the membrane were plotted (Figure 8). The second natural frequency and the third natural frequency had obvious bumps under 0.8 mT. The fourth natural frequency and the fifth natural frequency had bumps under 1.4 mT. The four curves had good linearity when the magnetic field was greater than 2 mT. By fitting the characteristic points with a magnetic field greater than 2 mT, it was found that all four curves had good linearity, with a sensitivity corresponding from the second natural frequency to the fifth natural frequency of approximately 84.2 kHz/mT, 212 kHz/mT, 166.7 kHz/mT, and 311.7 kHz/mT. The first natural frequency was calculated through previous simulations and was about 105 kHz/mT (Figure 4). Therefore, in theory, using the high-order mode of the graphene resonator can improve the sensitivity.

However, it should be noted that, in practical experiments, the amplitude gradually decreases with the increase in mode order [30]. Moreover, it is not easy to excite high-order modes, because the energy required for excitation is correspondingly increased. At present, two common driving methods include electrostatic force driving and laser driving. Due to the existence of parasitic capacitance of graphene resonators, electrostatic force driving struggles to excite very high resonant modes with sufficient signal strength using normal electrode configurations. For laser driving, not only is the experimental environment demanding, but the driving of higher resonance modes also requires shrinking the beam diameter of the laser to the nanometer level. Therefore, the excitation of high-order modes requires higher excitation energy, more expensive equipment, and a strict experimental environment. Recently, the fundamental modes (first- and second-order modes) were mostly used for sensing tests. Therefore, it is urgent to develop new methods to stimulate higher-order modes in the future.

Furthermore, since the magnetic sensor based on single-layer graphene and the magnetostrictive effect have not yet been reported, we chose two types of micro–nano devices for comparison. The first was a fiber-optic magnetometer based on graphene NEMS using superparamagnetic nanoparticles [35]. The reported resonant structure size was 10 μm, but graphene was multilayered; thus, the resonant frequency of the fundamental mode was higher than our simulation result. The quality factor measured in vacuum (10^−3^ Pa) reached 384. When the sensor was placed in a magnetic field, the strain caused by superparamagnetic nanoparticles in the graphene nanomechanical resonator changed, resulting in a shift in the resonant frequency. The sensitivity of the sensor was 104.5 Hz/mT. In contrast, our structure could improve the sensitivity by three orders of magnitude. The second device was based on Lorentz force [5]. Compared with these devices, this graphene nanomechanical resonator could increase the resonant frequency by three orders of magnitude (from 100 Hz to 100 kHz) and reduce the size of the core structure by about five orders of magnitude (mm^2^ to 10 μm^2^) on the premise of equal accuracy and similar signal reading mode.

In this work, the impact of changes in ambient temperature on the performance of the magnetic sensor was not considered. Chen et al. [32] studied the influence of ambient temperature on the resonance characteristics of suspended graphene resonators through experiments and found that temperature changes can affect the size of the resonant frequency. The resonant frequency decreased significantly with the increase in ambient temperature, while the tunability of the gate gradually increased. It could be seen that, if the ambient temperature changed greatly, the performance of the proposed magnetic sensor was weakened.

## 4. Conclusions

We analyzed and evaluated the feasibility of the proposed graphene resonance magnetic sensor from the perspective of finite element analysis. The sensing mechanism of the sensor was based on the frequency shift caused by the change of the axial stress at both edges of the graphene based on the magnetostrictive effect of Fe–Ga alloy. The results showed that, when the magnetic field *B*_0_ > 1 mT, the resonance frequency basically exhibited a linear change, and the sensitivity of the single-layer graphene resonance magnetic sensor could reach 105 kHz/mT. By further optimizing the size of the membrane, the substrate, and the Fe–Ga alloy, the maximum sensitivity could reach 834 kHz/mT, which is three orders of magnitude higher than previous resonant magnetic sensors based on magnetostrictive effect. Perhaps by changing the types of magnetostrictive materials, such as selecting TbFe, SmFe, and other alloy materials with large magnetostrictive coefficients, higher sensitivity can be obtained. This method theoretically opens up a new path for nano-opto-electromechanical magnetic sensing.

## Figures and Tables

**Table 1:** Material mechanical parameters.

Materials	Parameter	Value	Unit
Graphene [10]	Density	2200	kg/m^3^
	Poisson’s ratio	0.16	1
	Young’s modulus	1	TPa
Fe–Ga alloy [24,25,26,27]	Density	7600	kg/m^3^
	Elastic modulus	174.3	GPa
	Rigidity modulus	72.6	GPa
	Poisson’s ratio	0.2	1
	Saturation magnetization	60	kA/m
	Initial magnetic susceptibility	18	1
	Saturation magnetostriction	160	ppm
